# Myelopoiesis, metabolism and therapy: a crucial crossroads in cancer progression

**DOI:** 10.15698/cst2019.09.197

**Published:** 2019-07-01

**Authors:** Antonio Sica, Valentina Guarneri, Alessandra Gennari

**Affiliations:** 1 Department of Pharmaceutical Sciences, Università del Piemonte Orientale “Amedeo Avogadro”, via Bovio 6, Novara, Italy.; 2 Humanitas Clinical and Research Center, Via Manzoni 56, 20089 Rozzano, Milan, Italy.; 3 Department of Surgery, Oncology and Gastroenterology, University of Padova.; 4 Istituto Oncologico Veneto IOV I.R.C.C.S, Padova, Italy.; 5 Division of Oncology, Department of Translational Medicine, University of Eastern Piedmont, Novara, Italy.

**Keywords:** cancer, emergency myelopoiesis, tumor-associated macrophages, myeloid-derived suppressor cells, immune-metabolism, chemotherapy

## Abstract

Cancers promote immunological stresses that induce alterations of the myelopoietic output, defined as emergency myelopoiesis, which lead to the generation of different myeloid populations endowed with tumor-promoting activities. New evidence indicates that acquisition of this tumor-promoting phenotype by myeloid cells is the result of a multistep process, encompassing initial events originating into the bone marrow and later steps operating in the tumor microenvironment. The careful characterization of these sequential mechanisms is likely to offer new potential therapeutic opportunities. Here, we describe relevant mechanisms of myeloid cells reprogramming that instate immune dysfunctions and limit effective responses to anticancer therapy and discuss the influence that metabolic events, as well as chemotherapy, elicit on such events.

## INTRODUCTION

The bone marrow (BM) is a primary lymphoid organ that hosts hematopoietic progenitors and presides over hematopoiesis, which is defined as the production of all types of blood cells. Under steady-state conditions, hematopoiesis is a strictly regulated process that consists of a series of cell lineage commitments, encompassing sequential steps of differentiation, including transition of hematopoietic stem cells (HSCs) to lymphoid and myeloid precursors and subsequently to mature immune cells, necessary to maintain the physiological levels of circulating leukocytes [1]. In stress/pathological conditions (e.g. infection and cancer), signals derived from the HSCs niche modify the magnitude and composition of the hematopoietic output, a feature of immune regulation defined as ‘‘emergency'' hematopoiesis, to guarantee proper supply of both lymphoid and myeloid cells to increased demand [2]. In particular, in cancer altered myelopoiesis generates lineage-restricted hematopoietic progenitors, supporting the expansion of mature and immature myeloid cells endowed with tumor-promoting activities [3]. Within this scenario, tumor-associated macrophages (TAMs) and myeloid-derived suppressor cells (MDSCs) [4] are the main myeloid populations rising during tumor development and represent the final commitment of the protumoral reprogramming of the myeloid lineage. Moreover, Tie-2-expressing monocytes and tumor-associated neutrophils (TANs) infiltrate tumors, promoting angiogenesis and immunosuppression [5, 6].

“Emergency” myelopoiesis emerges in response to danger signals and is aimed at elimination of microbial threats, tissue repair and recovery of homeostatic conditions. Danger signals are sensed by myeloid cells through pattern recognition receptors (PRRs), able to detect pathogen-associated molecular patterns (PAMPs), conserved among entire classes of pathogens [7], as well as endogenous damage-associated molecular patterns (DAMPs), which are produced upon cellular stress and damage [8]. These events promote the production of cytokines and growth factors, which act through specific transcriptional programs that drive differentiation of myeloid cells.

Among these, interleukin-17A (IL-17A) promotes both granulocyte colony-stimulating factor (G-CSF)- and stem-cell-factor-mediated neutrophilia [9] and supports G-CSF-driven ‘‘emergency'' myelopoiesis[10]. IL-1 and IL-6 represent additional players in emergency myelopoiesis. In particular, IL-1 has been found to increase the proliferation and differentiation rate of HSCs [11] through induction of PU.1 and the consequent upregulation of both the macrophage colony-stimulating factor (M-CSF;*Csf1r*) and the granulocyte macrophage colony-stimulating factor (GM-CSF;*Csf2ra*) receptors. Interestingly, while chemotherapy-induced inflammation is a mechanism that reinforces aberrant myelopoiesis through the generation and expansion of MDSCs, IL-6 was confirmed to activate emergency myelopoiesis after myeloablation consequent to either cytotoxic treatment [12] or irradiation [13, 14]. Thus, the interplay between chemotherapy and inflammatory mediators critically controls the pathological expansion of tumor-promoting myeloid cells. Myelopoiesis is also critically affected by metabolism. In particular, cancer cells display increased glucose uptake and fermentation of glucose to lactate, even in the presence of completely functioning mitochondria. A major side effect of this event is immunosuppression, characterized by limited immunogenicity of cancer cells and restriction of the therapeutic efficacy of anticancer immunotherapy[15]. Correcting the pathological expansion of tumor-promoting myeloid cells during tumor growth appears therefore to be a promising strategy to improve anticancer responses and to generate more effective therapies.

## ORIGIN OF MYELOID SUPPRESSOR CELLS

In acute inflammation, notably during acute infections, myeloid progenitors expand and differentiate into activated pro-inflammatory monocytes, which eventually migrate into tissues where they mature to macrophages and dendritic cells (DCs) [16, 17]. Expansion of suppressor myeloid cells is peculiar to chronic inflammatory states (e.g. cancer, chronic infection and autoimmune disease), impairing the differentiation of myeloid progenitors into mature immune cells and leading to the expansion and accumulation suppressor myeloid cells, including MDSCs and TAMs [4]. Their expansion is an hallmark of cancer-immunosuppression and a major obstacle to anticancer treatments, since these populations exert a primary role in the organization of the immunosuppressive microenvironments [18, 19]. The detailed description of the mechanisms used by TAMs and MDCSs to promote tumor growth has been widely described in other works [3, 20] and goes beyond the scope of this review. Beyond being highly heterogeneous, TAMs and MDSCs are also highly plastic [21] and the surrounding microenvironment influences their functions to promote tumor development and to suppress immune responses through multiple mechanisms, including: depletion of metabolites critical for T cell functions, expression of immune checkpoint inhibitors, secretion of immunosuppressive molecules, production of reactive oxygen and nitrogen species, regulation of lymphocyte homing, expansion of regulatory T (Treg) cells [18]. In particular, TAMs are considered crucial orchestrators of cancer-related inflammation because they promote angiogenesis, immunosuppression, tissue remodeling and metastasis [20, 22]. The protumoral phenotype of TAMs is dictated by microenvironmental signals, which exploit the functional plasticity of macrophages, defined as the capacity to acquire a variety of functional states in response to different environmental stimuli. Indeed, *in vitro* macrophages activated by lipopolysaccharide (LPS) in the presence or absence of interferon-γ (IFNγ) (classically or M1/[LPS ± IFNγ]-activated) and those stimulated by IL-4 (alternatively or M2/[IL-4 ± IL-13]-activated) represent the extreme ends of a continuum of polarization states. Polarized macrophages differ in terms of receptors expression, cytokines/chemokines production, and effector functions. Although it is an oversimplification, the TAM's phenotype mostly resembles that of M2-like polarized macrophages. The phenotype of TAMs is strongly influenced by microphysiological conditions present in the surrounding microenvironment (e.g. hypoxia, interstitial hyperpression, low glucose levels) and molecularly and functionally distinct TAM subsets can simultaneously exist [23].

Along with TAMs, MDSCs are characterized by the capacity to suppress T cell functions and support tumor progression[3, 17]. These cells comprise at least two subsets: monocytic MDSCs (identified as CD11b^+^Ly6G^−^Ly6C^hi^ cells in mouse and CD11b^+^CD14^+^HLA−DR^low/−^CD15^−^ cells in human) and granulocytic MDSCs (PMN-MDSCs, identified as CD11b^+^Ly6G^+^Ly6C^lo^ cells in mouse and CD11b^+^CD14^−^CD15^+^ or CD11b^+^CD14^−^CD66^+^ cells in human) [24]. Despite the extensive literature on MDSCs, a consensus regarding the cellular definition of MDSC subsets has not yet been reached, as no specific markers exist to identify them unequivocally [24]. Nevertheless, due to the development of more sophisticated biochemical and gene expression profiling techniques, these cells are emerging as a pathologically activated population of immature myeloid cells. Therefore, on the basis of a panel of molecular, biochemical, and functional markers, an algorithmic approach to define cells as MDSCs has been proposed [17].

Globally, accumulation of myeloid progenitors and their differentiation to TAMs and MDSCs is the result of a process driven by cancer-related inflammation [25], involving: altered myelopoiesis; mobilization of myeloid precursors from the BM to periphery; recruitment of MDSCs and TAMs precursors into both secondary lymphoid organs and/or tumor tissues; functional diversion of myeloid cells in response to microenvironmental signals. This multistep process drives the reprogramming of myeloid cells towards a tumor-promoting phenotype and remotely controls the composition of the tumor-microenvironment. In support of this scenario, we recently showed that myeloid-specific expression of the retinoic-acid related orphan receptor (RORC1/RORγ) marks advanced cancer-inflammation [26] and expansion of circulating RORC1^+^ myeloid cells is associated with increased number of both immature suppressive cells (MDSCs) and TAMs [26]. We also reported that the M-CSF elevates the myeloid cell levels of nicotinamide phosphoribosyltransferase (NAMPT), the rate-limiting enzyme in the NAD salvage pathway, which acts as negative regulator of the CXCR4 retention axis of hematopoietic cells in the BM [27], hence promoting mobilization of myeloid cells to periphery. In agreement, NAMPT inhibition prevented MDSCs mobilization, reactivated specific antitumor immunity and enhanced the antitumor activity of immune checkpoint inhibitors [27].

**Figure 1:**
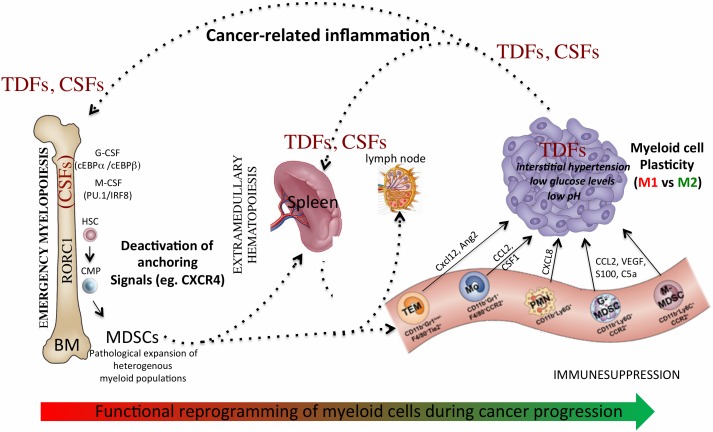
FIGURE 1: Myeloid cell reprogramming in cancer: a dynamic multistep process. Cancer-related inflammation promotes emergency myelopoiesis through production of colony stimulating factors, such as macrophage-colony stimulating factor (M-CSF), granulocyte-colony stimulating factor (G-CSF), granulocyte-macrophage- colony stimulating factor (GM-CSF). The transcription factor RORC1 is a key mediator of this myelopoietic response in emergency. Deactivation of anchoring signals, such as the retention axis CXCR4/CXCL12 promotes mobilization of myeloid cells to periphery and allows their accumulation to lymphoid organs, as well tumor tissues. Recruitment of myeloid cells into the tumor microenvironment expose these cells to additional signals and conditions that further boost their functional reprogramming towards a tumor-promoting phenotype. CSFs - Colony-stimulating factors, TDFs - tumor-derived factors, TEM - TIE2-expressing monocytes, PMN - polymorphonuclear cells, Mo – monocytes, MDSCs - myeloid-derived suppressor cells.

Additional evidences indicate that accumulation of TAMs and MDSCs in tumor tissues, as well as in metastasis, is guided by specific chemotactic pathways (eg. CCL2, M-CSF, CXCL2) [3, 28], suggesting possible therapeutic strategies to limit their recruitment and contribution to tumor growth. Lastly, microenvironmental signals and conditions, such as immunosuppressive cytokines (eg, IL-10, TGFβ) and hypoxia [29–31], dictate the final protumoral commitment of myeloid cells. Hence, this multistep process of myeloid cell reprogramming **(Figure 1)** may offer different levels of potential therapeutic interventions.

Similarly, activation and differentiation of DCs, the most potent antigen-presenting cells (APCs) of the immune system, is influenced by tumor growth, as well as by inflammatory and metabolic disorders [32]. Tumors alter host hematopoiesis and induce large numbers of immature DCs with immune suppressive properties. In addition, cancer cells produce immune suppressive factors (VEGF, IL-10, PGE2) that disable DC differentiation, maturation, migration, and functions [33]. Interestingly, while the 27 hydroxycholesterol (27HC) acts on HSCs via ERα to increase their proliferation and mobilization [34], oxysterols, that rise through enzymatic and non-enzymatic oxidation of cholesterol [35], interact with liver X receptors (LXRs) exerting an anti-inflammatory role on macrophages and DCs [36]. In agreement, oxysterols produced by tumor cells impair antigen presentation by inhibiting CCR7 expression on DCs [37]. Furthermore, DCs' immunogenicity is hampered by both TAMs and MDSCs, through the production of copious amount of indoleamine 2,3 dioxygenase 1 (IDO1) that converts tryptophan into kynurenines [38]. DCs differentiation is also affected by the gut microbiota, which may play a determinant role in the response to anticancer therapies. In particular, cancer chemotherapy and immunotherapy cause damage to intestinal epithelial barrier allowing bacteria translocation and/or changes in microbial composition, producing an adjuvant effect [39, 40]. Of relevance, Bifidobacterium species are associated with an anti-tumor response promoted by an enhanced activation of DCs, increased frequency of CD8+ T cells and greater response to anti-PD-L1 treatment [41]. Recent studies have also highlighted the role of energy metabolic pathways in the differentiation and function of myeloid cells [42]. In this regard, the deranged metabolic flux of cancer cells, characterized by aerobic glycolysis (Warburg effect) [47], results in the preferential conversion of pyruvate to lactate, which in turn impairs cytolytic T cell functions, and maturation of DCs [43].

## TRANSCRIPTIONAL CONTROL OF MYELOPOIESIS

The transcriptional basis guiding emergency myelopoiesis has only been partially clarified. Whereas C/EBPα appears to be a major regulator of ‘‘steady-state'' granulopoiesis [44], C/EBPβ [45] and Signal Transducer and Activator of Transcription 3 (STAT3) [46] promote expansion and maturation of neutrophils under emergency conditions. Furthermore, while in acute inflammation the C/EBPα interacts with the p50 NF-κB subunit to stimulate neutrophil production [47], C/EBPβ is a critical regulator of altered myelopoiesis in cancer bearers, contributing to the accumulation of MDSC and to the creation of an immunosuppressive environment [48]. Noteworthy, the BCR–ABL fusion protein activates emergency granulopoiesis by upregulating C/EBPβ, which in turn might support chronic myeloid leukemia [49].

Terminal macrophage differentiation is instead induced by M-CSF through activation of the transcription factors PU.1 and IRF8 [50]. We have recently shown that myeloid-specific expression of RORC1/RORγ marks advanced cancer stages [26] and orchestrates emergency myelopoiesis by suppressing negative (Socs3 and Bcl3) and promoting positive (C/EBPβ) regulators of granulopoiesis, as well as the key transcriptional mediators of myeloid progenitor commitment and differentiation to the monocytic/macrophage lineage (IRF8 and PU.1). Interestingly, IRF8 also functions as a "master" negative regulator of MDSC generation[51]. This plastic commitment of myeloid progenitors is further highlighted by the observation that, via HIF-1α activation, hypoxia redirects the differentiation of MDSCs toward tumor-associated macrophages, hence providing a mechanistic link between different myeloid suppressive cells in the tumor microenvironment [31].

Epigenetic modifications are also important regulators of myeloid cell functions. Recent studies demonstrate that chromatin-modifying enzymes could sense the macrophage's metabolic status (i.e. availability of acetyl-coenzyme A, S-adenosylmethionine, α-ketoglutarate (αKG), nicotinamide adenine dinucleotide and polyamines) to promote their transcriptional reprogramming and phenotypic changes [52]. Of relevance, Liu PS *et al.* recently demonstrated that αKG produced by glutaminolysis is an anti-inflammatory metabolite that augments M2 activation and controls metabolic reprogramming of M2 macrophages through the Jumonji domain containing-3 (Jmjd3)-dependent regulations [53]. Furthermore, epigenetic silencing of the retinoblastoma gene operated by histone deacetylase 2 (HDAC-2) drives the transdifferentiation of M-MDSCs into PMN-MDSCs in cancer [54].

## HOST METABOLISM AND MYELOPOIESIS

HSCs are mainly found in secured niches in the BM and are programmed for enforced quiescence. This “dormancy state” allows adult HSCs to be ready for quick and massive production of blood cells under emergency conditions, while limiting their proliferation to routinely production of blood cells. Therefore, under normal conditions, adult HSCs divide rarely to maintain low production of committed progenitor cells and refill the HSCs pool. However, they can rapidly and transiently proliferate in response to many inflammatory signals [55] such as acute infection, becoming rapidly activated for terminal differentiation and functional maturation, to exert their specialized immune functions against pathogens. Understanding how hematopoiesis is altered upon environmental stress is currently a major focus of research [56], addressed in preclinical models challenged with hematopoietic stresses such as infection or chronic inflammation. This metabolic influence has been extensively studied also under conditions of altered host metabolism, such as in the obese patients [57, 58]. Indeed, obesity-induced chronic inflammation is a key component in the pathogenesis of both insulin resistance and metabolic syndrome and is characterized by continuous production of proinflammatory cytokines that can lead to significant alteration in HSCs function and output [15, 59]. In difference to acute inflammatory stress, inducing a response that is quickly suppressed upon restoration of tissue homeostasis, failure to efficiently resolve inflammatory insults can have serious consequences for tissue maintenance and function. Indeed, in the context of chronic inflammation due to metabolic diseases such as obesity and type 2 diabetes, the inflammatory stress fails to resolve, leading to a persistent inflammatory state [15, 59].

Obesity, in fact, leads to a chronic inflammatory phenotype favoring the infiltration of activated immune cells into the adipose tissue and can be considered as a chronic pathological state associated with BM stresses [60]. In parallel, diabetes adversely impacts the mobilization capacity of HSCs by altering chemokine expression in the BM niche [61].

Of note, impaired blood system function is a common feature of these conditions, characterized by cytopenia in one or more lineages, anemia, thrombocytosis, suppression of lymphopoiesis, overproduction of myeloid cell populations that mediate further damage, or suppression of BM function [56, 62]. These data support the hypothesis that the enforced quiescence status prevents HSCs depletion during disease-induced chronic inflammation and suggest that the multipotent progenitor (MPP) compartment, which is responsible for everyday blood maintenance, may compensate ongoing needs, minimizing continued HSCs proliferation. In this perspective, it might also be considered that HSCs exhibit a finite replicative potential and chronic inflammation, or serial inflammatory episodes that induce HSCs proliferation, may create a maladaptive context, leading to HSCs decline and BM niche dysfunction. These findings have indeed raised new questions on the different impacts of inflammation on HSCs fate and function. The presence of a chronic inflammatory status associated with insulin resistance has also been linked with a higher risk of developing several types of cancer [63] by a number of epidemiological studies.

This association is biologically plausible as hyperinsulinemia induces proliferative tissue abnormalities due to the strong anabolic effect of insulin, resulting in the enhancement of DNA synthesis and cell proliferation. Furthermore, in patients with breast cancer (BC), higher circulating insulin levels have been found to be associated with an adverse outcome [64]. Recent evidence also indicates that the presence of insulin resistance is associated with a significantly worse prognosis in patients with advanced BC treated with chemotherapy [65]. Obesity itself has been hypothesized to impact response to chemotherapy, not only through metabolic perturbations such as the underlying insulin resistance status, hyperglycemia, adpokine production, and insulin-like growth factor (IGF)-1 system, but also affecting drug delivery, pharmacokinetics and transport. However, epidemiological studies in patients treated with chemotherapy have produced inconsistent results, with some showing an adverse effect and others a protective effect [66].

In cancer patients hematopoiesis is characterized by an increase in myeloid differentiation, which is believed to give an important contribution to the establishment of an immunosuppressive environment [67]. This is mainly due to the accumulation of MDSCs, that suppress adaptive immunity favoring cancer cell proliferation, tumor growth and survival, angiogenesis and metastasis. Under this hypothesis, the relationship between insulin resistance, obesity, metabolic impairment, with the underlying chronic inflammatory status and cancer needs to be extensively evaluated and approached in a multidisciplinary fashion. Indeed, the recent evidence [68] that patients with a higher Body Mass Index (i.e. overweight or obese) have an improved outcome if treated with immunotherapy for advanced tumors [69], indicates that the immune system, its targeting by cancer immunotherapy and patient metabolic status are closely connected. It is therefore critical to understand the potential effects of metabolic impairment on cancer related immunity, to improve treatment outcome with immuno-targeting.

## CELL METABOLISM AND MYELOPOIESIS

The relationship between metabolic alterations and the tumor-promoting functions of myeloid cells is further emerging as a crucial aspect of TAMs and MDSCs skewing toward pro-tumoral activities and their metabolic re-education appears as a new strategy to boost antitumor effector functions [15]. Within this scenario, the use of pharmacological agents to manipulate cell metabolism is an important step forward in the development of clinically relevant strategies. As an example, metabolites and metabolic regulators such as lactic acid, HIF1, c-Myc, adenosine monophosphate kinase (AMPK), and mTOR, which control metabolic reprogramming of immune cells and tumor cells, are being tested for targeting. Drugs targeting the lactate transporters MCT1 and MCT2 [70] and AMPK (e.g., metformin) are being evaluated for anti-tumor effects in preclinical models and in clinical trials [71].

Interestingly, besides affecting tumor cells, metformin has a direct effect on infiltrating immune cells: increasing CD8^+^ T cell recruitment, protecting them from apoptosis and exhaustion, increasing CD8^+^ memory T cells and providing a better response to anti-cancer vaccines [59]. Moreover, accumulating evidences indicate that AMPK, which is a central regulator of fatty acid, cholesterol, and glucose homeostasis, also skews macrophages polarization towards the M2 phenotype. More recently it was shown that in MDSCs, PPARγ plays a critical role in neutral lipid metabolism signaling controlled by lysosomal acid lipase (LAL) and that enhanced PPARγ activity impairs MDSCs-mediated proliferation and spreading of cancer cells [72]. Of note, both mouse and human tumor-infiltrating MDSCs, which accumulate during cancer progression, show a preferential increase of fatty acid uptake and fatty acid oxidation over glycolysis [73]. This metabolic profile is shared by M- and PMN-MDSCs subsets and dictates their immunosuppressive behavior inside the tumor [73]. Further, up-regulation of fatty-acid synthase (FASN) by M-CSF in tumor infiltrating myeloid cells was required for PPARβ/δ-dependent expression of immunosuppressive and pro-angiogenic genes (e.g. IL-10, Arg1 and VEGF) and consequent promotion of tumor progression [74], in a model of Lewis lung cancer.

In this perspective, the antitumor effect of metformin was recently evaluated in two prospective, randomized trials, in non-diabetic women affected by early and advanced BC. The first one was a window of opportunity, double-blind, randomized study in early BC patients, candidate to surgery: in this setting, the administration of metformin single agent for four weeks before surgery, did not impact tumor proliferation, compared to baseline levels, in the overall patient population. However, a significant effect was seen in insulin-resistant patients [75]. In the metastatic setting, the addition of metformin to first-line chemotherapy as compared to chemotherapy alone, did not improve progression free survival or overall survival. Of note, patients receiving the combination of metformin and chemotherapy experienced significantly lower severe neutropenia as compared to chemotherapy alone, suggesting a protective effect on BM toxicity [65]. A large adjuvant trial on non-diabetic women with early BC comparing metformin with matching placebo in terms of disease-free survival (DFS) is currently ongoing.

## CHEMOTHERAPY EFFECTS ON MYELOPOIESIS AND IMPLICATION FOR IMMUNOTHERAPY

Malignant transformation and progression cannot occur without the aid of host-derived factors. The interplay among tumor cells, stroma and immune system cells contributes to the shaping of tumor microenvironment where reprogrammed non-cancer cells sustain angiogenesis, cell growth, diversion and skewing of adaptive response. As previously discussed, the myeloid compartment plays a key role in this tumor reconditioning, which results in the generation of myeloid cells with special immunosuppressive properties. Of note, atypical myelopoiesis has also been described in hematologic malignancies including multiple myeloma, leukemias and lymphomas. In this perspective, acute and chronic myeloid leukemia can be considered the most extreme examples of deregulated myelopoiesis [76].

Chemotherapy represents an important component of the therapeutic armamentarium against cancer. More recently, restoring immune response with immune check-point inhibitors has emerged as an efficient treatment for several cancer types. Even though apparently counterproductive, the combination of these two strategies resulted in clinically meaningful results [77–79]. Indeed, chemotherapy has been historically viewed as immunosuppressive mainly because of its lymphodepleting effect. However, chemotherapy effects on the immune system are extremely complex. Most preclinical studies support chemotherapy-induced inflammation as a mechanism to reinforce aberrant myelopoiesis, through the generation and expansion of MDSCs. This effect is suggested as a counter-regulatory adaptation to prevent unnecessary damage from a chemical insult [80]. Enhancement of MDSCs suppressive activity is described with doxorubicin and with high-dose cyclophosphamide, among others [81]. In contrast, other preclinical data have shown that a number of cytotoxic agents, such as gemcitabine, docetaxel and 5-fluorouracil, can induce MDSCs apoptosis [82–84]. Cyclophosphamide can be considered the paradigm of the complexity of the interplay between chemotherapy and the immune system, since both immuno-stimulating effects as well as induction of immunosuppressive cells have been described with this cytotoxic agent [85]. Indeed, MDSCs accumulation was reported by several authors [86, 87]. However, when administered at low dose, cyclophosphamide causes Treg cell depletion, augmenting antitumor response and increasing expression of DC maturation markers [88, 89]. Data from clinical studies prospectively evaluating the effects of chemotherapy on MDSCs are scanty, and the results somewhat conflicting. Diaz *et al.* [90] evaluated 17 stage II-III BC patients treated with adjuvant dose-dense doxorubicin-cyclophosphamide (AC) followed by dose dense paclitaxel [91]. A significant increase in MDSCs was described after dose-dense AC. More recently, in 24 BC patients undergoing neoadjuvant sequential chemotherapy, a significant increase in granulocytic-MDSC was reported after AC therapy, with subsequent decrease to near baseline levels during paclitaxel treatment [90]. PMN-MDSCs levels at the last draw were numerically lower in patients with pathologic complete responses (pCRs) versus patients with no pCR. However, these data are far from being conclusive, considering the limited sample size, the inclusion of heterogeneous patient population with respect to hormone receptor and HER2 expression, as well as the use of G-CSF. Indeed, G-CSF is one of the key drivers of aberrant expansion of myeloid cells, and the common use of exogenous G-CSF in cancer patients undergoing chemotherapy might play a role in the immunosuppressive status induced by the tumor. The largest study so far included 56 patients with locally advanced BC undergoing neoadjuvant therapy with sequential AC followed by docetaxel+/- capecitabine [92]. In contrast to the findings of the other two trials, monocytic and granulocytic MDSCs were significantly reduced after both four and eight courses of chemotherapy, irrespective of response. Interestingly, circulatory levels of Treg were significantly associated with pathologic response.

In patients with non-small cell lung cancer treated with first-line platinum-based chemotherapy, higher M-MDSCs significantly correlated with worse outcome. However, dynamic changes of MDSCs during chemotherapy were not evaluated [93]. In 23 patients with advanced colorectal cancer, decreased levels of MDSCs were observed after therapy with 5-fluorouracil combined with oxaliplatin (FOLFOX), while persistent increase of MDSCs was reported after 5-fluorouracil combined with Irinotecan (FOLFIRI) [94]. As a whole, these data suggest that chemotherapy can impact on the tumor microenvironment by promoting antitumor immune response, or by inducing MDSCs that counter-regulate immune response.

Immunotherapy is now established as a ground-breaking strategy in several tumors; however, a significant proportion of patients does not respond or even experience hyperprogression. Taking into account the costs, as well as the potential side effects of these treatments, adequate patient selection is highly needed. Tissue biomarkers are promising, but not suitable for dynamic evaluation. In this context, circulating immune-related biomarkers are particularly attractive. Peripheral blood mononuclear cells phenotyping is considered a dynamic marker to evaluate pre-existing immunity that could affect outcome and sensitivity to treatments. MDSCs levels have been associated with prognosis in ipilimumab-treated patients. In particular, on-treatment high levels of CD14^+^/IL4Rα^+^ MDSCs were negative independent factors of reduced overall survival in a cohort of melanoma patients treated with ipilimumab [95].

Prospective MDSCs and TAMs evaluation in trials of chemo-immunotherapy could elucidate the mechanisms underlying different tumor behaviors upon treatment exposure. Moreover, identifying the drivers of treatment resistance can be helpful to select potential targets to restore antitumor immune response.

## CONCLUSIONS

Solid tumors are composed by cancer cells, stroma and a variety of infiltrating immune cells, which establish reciprocal relationships that dictate the clinical outcome. Despite this evidence, the mechanisms leading to accumulation of tumor-promoting myeloid cells in the tumor microenvironment are far from being understood, though might likely be the sum of sequential alterations targeting: differentiation of myeloid progenitors and their mobilization to periphery; their recruitment to both secondary lymphoid organs and tumor tissues; their functional diversion in response to microenvironmental signals and conditions. Efforts are being made to characterize the immunostimulatory properties of chemotherapeutic agents and how they can be best combined with immune checkpoint inhibitors and new evidence indicates that chemotherapeutics (e.g. paclitaxel acting as TLR4 agonist) [96] can restore the anticancer activity of TAMs and improve the clinical efficacy of immune checkpoint inhibitors. In this perspective, while the tumor microenvironment has been considered as a suitable target for therapeutic interventions, new studies should carefully evaluate the impact of therapies on the quality and extent of the hematopoietic response, aiming to differentially target multiple levels of the tumor-promoting reprogramming of myeloid cells. The analysis of the effects and mechanisms elicited by different chemotherapeutics, dose and timing of administration, as well as their interplay with metabolic traits, seems therefore crucial to establish the drivers of myelopoietic alterations associated with tumor progression and for a correct stratification of patients, in order to achieve a rational combination that can activate synergism between chemotherapy and immunotherapy.

## Abbreviatons:

αKG: – α-ketoglutarate,
AMPK: – adenosine monophosphate kinase,
BC: – breast cancer,
BM: – bone marrow,
DC: – dendritic cell,
G-CSF: – granulocyte colony-stimulating factor,
HSC: – hematopoietic stem cell,
M-CSF: – macrophage colony-stimulating factor,
MDSC: – myeloid-derived suppressor cell,
NAMPT: – nicotinamide phosphoribosyltransferase,
pCR: – pathologic complete response,
TAM: – tumor-associated macrophage,
Treg: – regulatory T cell.
